# Evolution of the hypoxic compartment on sequential oxygen partial pressure maps during radiochemotherapy in advanced head and neck cancer

**DOI:** 10.1016/j.phro.2021.01.011

**Published:** 2021-02-11

**Authors:** Marta Lazzeroni, Ana Ureba, Nicole Wiedenmann, Nils H. Nicolay, Michael Mix, Benedikt Thomann, Dimos Baltas, Iuliana Toma-Dasu, Anca L. Grosu

**Affiliations:** a Department of Physics, Stockholm, Sweden; b Skandion Clinic, Uppsala, Sweden; c Department of Radiation Oncology, Medical Center, Medical Faculty Freiburg, German Cancer Consortium (DKTK) Partner Site Freiburg, Freiburg, Germany; d Department of Nuclear Medicine, University Medical Center, Freiburg, Germany; e Department of Oncology and Pathology, Karolinska Institutet, Stockholm, Sweden

**Keywords:** Hypoxia, FMISO PET, pO_2_, HNSCC, Radiochemotherapy

## Abstract

**Background and purpose:**

Longitudinal Positron Emission Tomography (PET) with hypoxia-specific radiotracers allows monitoring the time evolution of regions of increased radioresistance and may become fundamental in determining the radiochemotherapy outcome in Head-and-Neck Squamous Cell Carcinoma (HNSCC). The aim of this study was to investigate the evolution of the hypoxic target volume on oxygen partial pressure maps (pO_2_-HTV) derived from ^18^FMISO-PET images acquired before and during radiochemotherapy and to uncover correlations between extent and severity of hypoxia and treatment outcome.

**Material and methods:**

^18^FMISO-PET/CT images were acquired at three time points (before treatment start, in weeks two and five) for twenty-eight HNSCC patients treated with radiochemotherapy. The images were converted into pO_2_ maps and corresponding pO_2_-HTVs (pO_2_-HTV_1_, pO_2_-HTV_2_, pO_2_-HTV_3_) were contoured at 10 mmHg. Different parameters describing the pO_2_-HTV time evolution were considered, such as the percent and absolute difference between the pO_2_-HTVs (%HTV_i,j_ and HTV_i_-HTV_j_ with i,j = 1, 2, 3, respectively) and the slope of the linear regression curve fitting the pO_2_-HTVs in time. Correlations were sought between the pO_2_-HTV evolution parameters and loco-regional recurrence (LRR) using the Receiver Operating Characteristic method.

**Results:**

The Area Under the Curve values for %HTV_1,2_, HTV_1_-HTV_2_, HTV_1_-HTV_3_ and the slope of the pO_2_-HTV linear regression curve were 0.75 (p = 0.04), 0.73 (p = 0.02), 0.73 (p = 0.02) and 0.75 (p = 0.007), respectively. Other parameter combinations were not statistically significant.

**Conclusions:**

The pO_2_-HTV evolution during radiochemotherapy showed predictive value for LRR. The changes in the tumour hypoxia during the first two treatment weeks may be used for adaptive personalized treatment approaches.

## Highlights

• Repeated PET imaging of hypoxia may be pivotal in radiotherapy outcome prediction.
• Oxygen partial pressure maps can be non-linearly derived from radiotracer uptake.
• The hypoxic target volume evolution in extension and severity can be determined.
• The first two treatment week parameters have potential for outcome prediction.
• Information may be used for treatment adaptation personalised strategies.

## 1 Introduction

Functional imaging with Positron Emission Tomography (PET) in radiotherapy is rapidly evolving from tumour stage assessment and tumour evolution monitoring towards treatment outcome prediction [1], [2]. Tumour microenvironment, in general, and tumour hypoxia, in particular, have been looked as the main determinants of cell radioresistance and among the leading causes of radio(chemo)therapy response failure for solid malignant tumours such as head and neck (H&N) cancer [1], [2].

Hypoxia-specific radiotracers, e.g. ^18^F-fluoromisonidazole (^18^FMISO), allow not only visualising hypoxic areas, but offer also a way to characterise quantitatively the microenvironment. The use of conversion functions of radiotracer uptake into oxygen partial pressure maps (pO_2_ maps) is further advantageous because it enables to theoretically determine the hypoxic target volume (pO_2_-HTV) dose escalation level for achieving a desired Tumour Control Probability (TCP) [3], [4], [5].

The framework for tumour hypoxia characterisation and dose boost strategy based on pO_2_ maps has been proposed [3], [4], [5]. However, it remains uncertain at which time point(s), before/during treatment, the ^18^FMISO-PET image(s) should be acquired for optimal pO_2_-HTV characterisation, related dose (de)escalation strategy selection, and outcome prediction. It is well-known that re-oxygenation takes place during the radio(chemo)therapy and several studies have shown that the HTV may change during radiotherapy in severity and location [2], [6], [7], [8], [9], [10], [11], [12]. Thus, one single time point evaluation may be less effective for treatment outcome prediction [7]. While many studies have focused on the pre-treatment information [13], [14], [15], a few have looked at different time points and tried to assess which of the tumour parameters are most significant for treatment outcome prediction [16], [17].

The correlation between several ^18^FMISO PET-derived image parameters and local progression-free-survival was studied by Zips et al. [7] in 25 Head-and-Neck Squamous Cell Carcinoma (HNSCC) patients. The authors found a stronger association, compared to baseline, at early time points during treatment, i.e. week one (8–10 Gy) and two (18–20 Gy). In a validation study of [7], Löck et al. [18] found that image parameters extracted after one-two treatment weeks were able to select patients developing loco-regional recurrence (LRR). Furthermore, the authors found that residual tumour hypoxia, defined as the ratio of the HTV in week two and prior treatment, was prognostic for LRR. Wiedenmann et al. [2] analysed 16 HNSCC patients and found that the tumour-to-background ratio was able to stratify the patients in responders (R) and non-responders (NR) to treatment, in terms of local recurrence free survival, both at baseline and after two treatment weeks, with a stronger association at the second time point. Other results on repeated hypoxia PET measurements in HNSCC are reviewed by Stieb et al. [16]. A radiomics study of hypoxia PET imaging by Sörensen et al. evidenced that textural features on ^18^FMISO-PET scans before treatment, in week two and the change of features during treatment, were able to predict overall survival [17].

While most studies analysed the HTV seeking for the optimal time point at which a higher correlation would be found between extracted image parameters and treatment outcome [16], we focused on the assessment of the global trend in the HTV evolution, concerning both its extension and severity. Therefore, we considered the initial tumour characteristics as a reference point guiding its evolution, rather than looking at absolute tumour parameters. Furthermore, we investigated the changes in the parameters characterising the tumour after converting the images into pO_2_ maps, because the tumour oxygenation is a well-known key factor affecting the radiosensitivity [19] and the relationship between the radiotracer uptake and pO_2_ is not linear [3], [4], [5]. Specifically, the aims of this study were (I) to assess whether the gradient in tumour parameters between two time points would suffice for stratifying the patients in R and NR or if more time points would be necessary and (II), in case only two time points would suffice, to assess for which of the two paired parameters the correlation would be stronger.

## 2 Materials and methods

### 2.1 Treatment protocol and PET/CT image acquisition

Twenty-eight patients with locally advanced HNSCC were treated with concomitant radiochemotherapy at the University Medical Center Freiburg (Germany). The Gross Tumour Volume including both the primary tumour and the lymph nodes (GTV_all_) was delineated by experienced clinicians using Computed Tomography (CT), Magnetic Resonance Imaging (MRI), and ^18^Fluorodeoxyglucose (^18^FDG)-PET images. On the ^18^FDG-PET image a threshold on the 40% of the maximum Standardised Uptake Value (SUV) was considered. The radiochemotherapy protocol consisted of a conformal Intensity Modulated Radiation Therapy treatment with total dose of 70 Gy in five fractions per week over seven weeks, and three cycles of chemotherapy with cisplatin (100 mg/m^2^). Mean follow-up time was 20 ± 17 months (range 3–61, median 13). Minimum follow-up time for responders to treatment with respect to LRR was one year. The prospective imaging study had the approval of the Ethics Committee of institutional review board (DRKS00003830).

Patients were imaged three times with ^18^FMISO-PET/CT: before the start of the treatment (−6 ± 3 days), in week two (11 ± 4 days, 18 ± 6 Gy), and in week five (33 ± 6 days, 49 ± 8 Gy). Days were counted from the start of radiotherapy (t = 0). The ^18^FMISO-PET image acquisition started 160 min after the injection of 300 MBq of radiotracer (303 ± 21 MBq) with one bed position covering the whole H&N region. Patients were imaged in radiotherapy position with a H&N mask. Main patient characteristics of interest for the analysis are reported on Table 1 and on Table A of the Supplementary Material.

**Table 1:** Clinical and demographic patient characteristics.

Patient Characteristic	Value
*Age [years]*	
Average ± SD, median	59.5 ± 8.3, 60.5

*Sex*	
Male	27 (96%)
Female	1 (4%)

*Karnofsky performance index*	
100%	12 (43%)
90%	11 (39%)
80%	4 (14%)
70%	1 (4%)

*Smoking status*	
Positive smoking status	22 (80%)
Negative smoking status	6 (20%)

*Tumour location*	
Oral cavity	2 (7%)
Oropharynx	9 (32%)
Hypopharynx	8 (29%)
Larynx	4 (14%)
Multi-level	5 (18%)

*Tumour extent*	
T1	1 (3%)
T2	2 (7%)
T3	8 (29%)
T4	17 (61%)

*Nodal status*	
N0	4 (14%)
N1	1 (4%)
N2a	0 (0%)
N2b	5 (18%)
N2c	18 (64%)

*Grading*	
G1	0 (0%)
G2	16 (57%)
G3	12 (43%)

*HPV status*	
HPV Positive	6 (21%)
HPV Negative	22 (79%)

### 2.2 Image analysis

PET data were reconstructed using an ordered-subset expectation-maximization algorithm to voxels of (2 × 2 × 2) mm^3^. Scatter and attenuation corrections were performed on the images. The ^18^FMISO-PET/CT scans acquired at the three time points were co-registered to the planning CT (CT_plan_) in a research version of the treatment planning system RayStation (RaySearch Laboratories AB, Sweden). The CT_plan_-to-CT registration consisted of a rigid registration based on the bony anatomy followed by a hybrid deformable registration. The latter combines image information with anatomical information as provided by contoured image sets and uses as controlling region the external patient contour aiming at anatomical structure integrity [20].

The ^18^FMISO-PET uptake was converted into pO_2_ distribution by applying a sigmoid conversion function at voxel level [21], [3], [4], [5]:

(1) $$pO_{2}=\frac{c(a-Uptake\left(r\right))}{b+Uptake\left(r\right)-a}$$

where a, b, c are reaction-specific parameters equal to 10.9, 10.7, and 2.5 mmHg, respectively. The parameter *Uptake* in Eq. (1) was calculated as follows: the voxel values in the ^18^FMISO-PET images were divided by the average value in a Well Oxygenated Volume (WOV) and the results were multiplied by the tracer uptake predicted by the conversion function for the assigned pO_2_ in the WOV. The deep neck muscle volume, delineated by an expert radiologist, was chosen as WOV [2], [8] with an assigned pO_2_ equal to 30 mmHg [22], [23]. The pO_2_-HTV was then delineated by automatically thresholding the pO_2_ maps at 10 mmHg and intersecting with the clinical target volume (CTV).

The HTV time evolution was evaluated by delineating the pO_2_-HTVs in the three sets of pO_2_ maps (HTV_1_, HTV_2_, HTV_3_) corresponding to the three sequential ^18^FMISO-PET images and considering the percent difference between them, %HTV*_i,j_*:

(2) $$\%HTV_{i,j}=\;\left(\mathrm{HT}V_{i}-HTV_{j}\right)/HTV_{i}\cdot 100$$

with i and j = 1, 2, 3. The variation in time of the percent hypoxic fraction, defined as %HF = HTV/CTV * 100, was also analysed.

The global variation of the pO_2_-HTVs in time was then analysed by considering all the three time points in the following cases: (a) a global linear regression analysis was performed for the pO_2_-HTVs evolution in time for the two subgroups of R and NR and the two resulting slopes were scored, (b) the slope of the linear regressions fitting the pO_2_-HTVs in time for each of the patients was scored.

### 2.3 Statistical analysis

The non-parametric Wilcoxon signed-rank test for dependent samples [24] was used to compare minimum oxygen partial pressure level in pO_2_-HTV between paired pO_2_ maps (min pO_2(1_*_,_*_2)_, min pO_2(1_*_,_*_3)_, min pO_2(2_*_,_*_3)_) and volume of pO_2_-HTV in paired pO_2_ maps (HTV_1_*_,_*_2_, HTV*_1__,_*_3_, HTV_2_*_,_*_3_) at the three different time points for the same patient dataset. The non-parametric Mann-Whitney signed-rank test for independent samples [25] was used when comparing image-extracted parameters for R and NR to treatment. The Receiver Operating Characteristic (ROC) method was used to seek for correlations between LRR and the quantities of interest mentioned above. The optimal criterion of the ROC analysis [26] was scored together with the Area Under the Curve (AUC) and corresponding p-values. A p-value ≤0*.*05 was considered statistically significant. The software MedCalc was used for the statistical analysis (MedCalc Software, Belgium).

## 3 Results

**Fig. 1:**
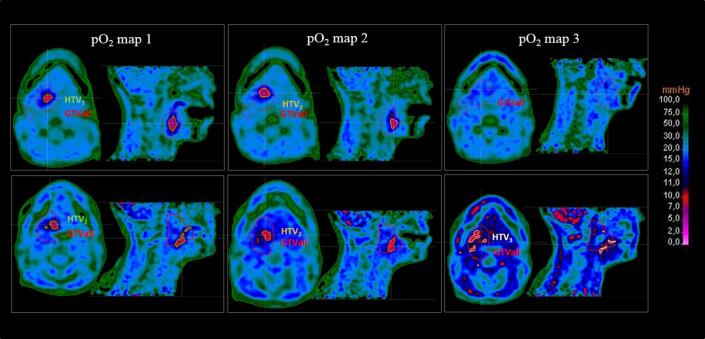
Representative oxygen partial pressure, pO_2_, maps at the three different time points (before treatment: pO_2_ map 1, at week 2: pO_2_ map 2, and at week 5: pO_2_ map 3) derived from ^18^FMISO PET scans are shown for a patient with non-recurrent tumour (patient 13, upper panel) and for a patient developing recurrence (patient 12, lower panel). Hypoxic target volumes (HTV, corresponding to a 10 mmHg threshold) and total Gross Tumour Volume (GTV_all_, given by the union of the primary GTV and the GTV of the lymph nodes) are contoured in the images.

Twenty-one out of twenty-eight patients (75%) were found to have a pO_2_-HTV at the first time point. The number of hypoxic cases then reduced to 18 (64%) and to 9 (32%) at the second and third time points, respectively. Of those 21 initial hypoxic cases, 13 (62%) had also lymph node involvement and 3 (14%) showed presence of hypoxic volumes only in the lymph node area. Representative pO_2_ maps are shown in Fig. 1 for two patients: patient 13 (upper panel) and patient 12 (lower panel), respectively, R and NR to treatment in terms of LRR. HTVs were contoured in the figure together with GTV_all_. Patient 13 (R) had %HTV_1_*_,_*_2_ = 43%, %HTV_1_*_,_*_3_ = 100% and %HTV_2_*_,_*_3_ = 100% with a %HF evolving in time from 1.9% to 1.1% and, finally, 0%. Patient 12 (NR) had a slightly smaller hypoxic fraction from the start %HF_1_ = 1.8% (as well as a slightly smaller HTV_1_ equal to 3.6 cm^3^ versus 4.2 cm^3^ of patient 13) which evolved in time as follows: %HF_2_ = 1.2%, %HF_3_ = 2.3%. The percent difference between the volumes was %HTV_1_*_,_*_2_ = 29%, %HTV_1_*_,_*_3_ = −34% and %HTV_2_*_,_*_3_ = −89%, thus indicating an initial regression of the hypoxic core followed by a final progression.

The non-parametric Wilcoxon signed-rank test for the minimum pO_2_ values and the HTVs at the different time points resulted in: min pO_2(1_*_,_*_2)_ with p = 0.01, min pO_2(1_*_,_*_3)_ with p = 0.0001, min pO_2(2_*_,_*_3)_ with p = 0.02; HTV_1_*_,_*_2_ with p = 0.002, HTV_1_*_,_*_3_ with p = 0.0002, HTV_2_*_,_*_3_ with p = 0.01.

**Fig. 2:**
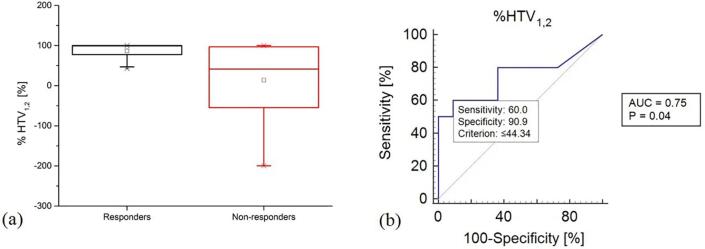
Box plot (a) and Receiver Operating Curve (ROC) (b) of the percent difference, %HTV_1_*_,_*_2_, between the hypoxic target volumes scored at the first and second time point on oxygen partial pressure maps (10 mmHg threshold). The ROC curve is calculated by considering the correlation with tumour loco-regional recurrence.

Box plot of %HTV_1_*_,_*_2_ (Eq. (2)) calculated on the first and second pO_2_ maps showed that recurrent tumours both had increased and decreased HTVs at the second week time point (range [−200,100]%, mean 14%, median 42%), while all the non-recurrent tumours showed a decrease in their volume (range [43,100]%, mean 87%, median 99%) (Fig. 2a). The non-parametric Mann Whitney signed-rank test comparing the %HTV_1_*_,_*_2_ in the R and NR groups had a p-value of 0.05.

Results of the ROC analysis correlating %HTV_1_*_,_*_2_ with LRR had AUC value equal to 0.75 (p-value of 0.04, optimal criterion ≤44.3% with sensitivity = 60% and specificity ≈ 91%) (Fig. 2b). Absolute values of the difference between the HTVs at the first and second time points (HTV_1_ − HTV_2_ in cm^3^), and at the first and third time points (HTV_1_ − HTV_3_ in cm^3^), had AUC value equal to 0.73 (p-value of 0.02, optimal criterion ≤1.26 cm^3^ with sensitivity = 80% and specificity = 62%) and AUC value equal to 0.73 (p-value of 0.02, optimal criterion ≤1.26 cm^3^ with sensitivity = 80% and specificity = 62%), respectively. The other combinations of percent or absolute differences between HTVs at the different time points did not have statistically significant AUC values.

**Fig. 3:**
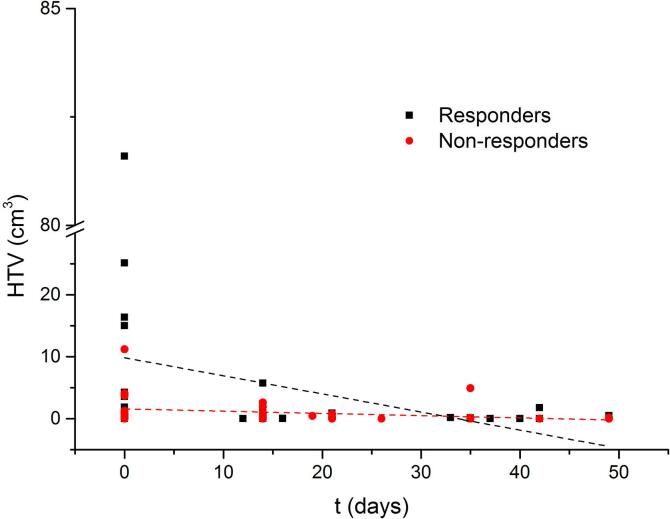
Global variation of the hypoxic target volume, contoured on obtained pO_2_ maps using a 10 mmHg threshold, as a function of time for the three available time points for the entire group of responders (full black squares) and non-responders (full red circles). Time in days is calculated from the start of the radiation treatment. The two linear regression curves are shown in the figure for recurrent (red dashed line) and non-recurrent (black dashed line) tumours. (For interpretation of the references to colour in this figure legend, the reader is referred to the web version of this article.)

**Fig. 4:**
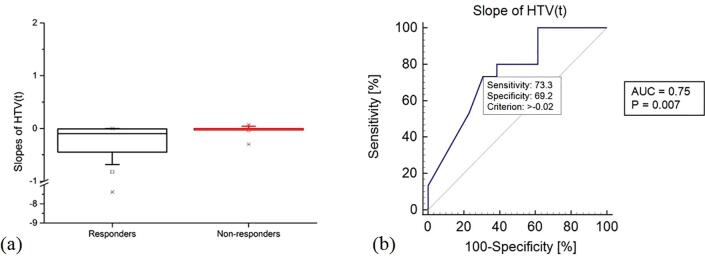
Box plot (a) and Receiver Operating Curve (ROC) (b) for the slope of the fitting linear regression curve of the hypoxic target volumes as a function of time for each individual patient in the data set. The ROC curve is calculated by considering the correlation with tumour loco-regional recurrence.

The global linear regression analysis of the pO_2_-HTVs in time for R and NR resulted in a slope of −0.29 ± 0.15 for R and −0.04 ± 0.02 for NR (Fig. 3). The slopes of the linear regression curves evidenced the global decrease of the HTV for the R group as opposite to the NR group where the permanence of the HTV in time was apparent. These global trends were further corroborated by the results in Fig. 4a.

Box plots of the slopes of the linear regression curve fitting the pO_2_-HTVs in time for each individual patient in the R and NR subgroups showed that: for NR, the box plot was restricted around the 0 slope (range [−0.30, 0.07], mean −0.03, median −0.002), and, for R, a higher variability was found (range [−7.38, 0], mean −0.82, median −0.10), which spanned only on the negative portion of the scale (Fig. 4a). The non-parametric Mann-Whitney signed rank test comparing the slopes in the R and NR groups had a p-value of 0.02. Moreover, when seeking for correlation between the slope and LRR, the ROC analysis (Fig. 4b) resulted in an AUC equal to 0.75 (p-value = 0.007, optimal criterion >−0.02 with sensitivity = 73% and specificity = 69%). Additional comparisons and predictive capabilities of ^18^FMISO-PET-derived and pO_2_-derived quantities are reported on Tables B and C in the Supplementary Material.

## 4 Discussion

In this study, we investigated the global time evolution of the HTV on pO_2_ maps derived from ^18^FMISO-PET images. The slope of the linear fit of the pO_2_-HTV in time, the percent and absolute difference in pO_2_-HTV before treatment and during week two, as well as the absolute difference in pO_2_-HTV before treatment and during week five were statistically significant for outcome prediction.

Obtained results indicate that longitudinal PET imaging of hypoxia may be pivotal in radio(chemo)therapy for outcome prediction assessment at an early treatment stage. In particular, the statistical significance of the gradient between pre-treatment and week two image parameters (Fig. 2) confirmed the expectations from previous investigations on longitudinal PET imaging of hypoxia, also indicating that crucial information is retained early during treatment [2], [7], [17], [18]. On a broader perspective, studies focused on ^18^FDG-PET images have also shown that week two may carry fundamental information for outcome prediction [27], while week three may not [28]. Even though week three may still conserve important information about the tumour dynamics, the relatively poor PET resolution may not allow distinguishing remaining niches of tumour cells from the background uptake. Similar considerations may also explain why, in this study, the difference in pO_2_-HTV pre-treatment and on week five was only significant in absolute difference and not in percent difference.

The actual absolute volume of pO_2_-HTV scored at the three different time points was not statistically significant in differentiating R from NR (cfr. Fig. 3), instead the capability in dichotomizing the considered dataset laid in the volume variations in time (Fig. 2). To note that, while in other studies [2], [8] the HTV was defined as a Sub-Volume of the GTV (HSV), which is, on its turn, defined based on morphological changes evidenced from MRI, CT images or on the combination of anatomical and functional changes with the use of combined ^18^FDG-PET/CT/MRI scans; in this study, the full extension of the HTV volume within the CTV was considered. In fact, tumour hypoxia may not be restricted to the anatomical information from morphological images, since the functional information may reveal earlier than the anatomical one [27]. Moreover, even when functional and metabolic information extracted from combined ^18^FDG-PET/CT/MRI images is included in the GTV definition, ^18^FMISO-PET and ^18^FDG-PET information may show poor/partial correlation with each other, which may question the HTV delineation as a GTV sub-compartment [29]. For completeness, the authors have also considered the above mentioned HSV by further intersecting the HTV with the GTV, however, no changes in the statistical significance of the HSV results were obtained.

When several images acquired at different time points are considered and delineated volumes are compared, questions may arise on the type and quality of the image registration. The hybrid algorithm for deformable registration was extensively validated and it performed well in comparison to other algorithms [20]. However, in this study, the HTVs were delineated on the pO_2_ maps derived from ^18^FMISO-PET images (rigidly registered to CT_plan_) and no deformable registration was, therefore, necessary. In fact, the only Region Of Interest (ROI) that may have needed a deformable registration between the ^18^FMISO-PET and CT_plan_ would have been the ROI of the neck muscle used for calculating the average tracer uptake (cfr. Eq. (1)). Nevertheless, for this ROI, the lack of deformable registration between the ^18^FMISO-PET and CT_plan_ was not a matter of concern for two main reasons: (a) the actual neck-ROI was merely used for calculating an average uptake on a rather extensive region (thus, no major changes would have been expected from a slight modification of the volume (see point (b)), (b) the vector displacement field of the deformable image registration between CT_plan_ and the CT of the ^18^FMISO-PET in the area around the neck-ROI was found small and comparable with the uncertainties of deformable registration algorithms [20], [30]. The prediction of the tumour response to radio(chemo)therapy at an early time point is a necessary step towards the development of treatment adaptation strategies by dose boosting or, possibly, dose de-escalation. In this sense, obtained results pointing at the second week as the significant one to evaluate the treatment outcome are promising, since a treatment alteration at a very early time point would be allowed by using hypoxia dose painting methods based on pO_2_ maps [3], [4], [5] and algorithms accounting also for the possible geographical de-localization of the HTV in longitudinal PET images [31]. Furthermore, it may be mentioned that the assessment of hypoxia with pre-treatment dynamic ^18^FMISO-PET scans has shown to be promising for outcome prediction assessment and, eventually, dose painting [32]. However, as compared to pre-treatment dynamic ^18^FMISO-PET, repeated imaging would help to rule out any possible geographical HTV miss that may occur during the course of treatment [8]. In this sense, dynamic ^18^FMISO-PET repeated during treatment and possibly coupled with uptake-to-pO_2_ conversion might increase the outcome prediction capability. It is difficult to assess *a priori* which method would be more advantageous and an answer may only be found on large-scale clinical trials.

In conclusion, this study showed that the evolution of the hypoxic compartment during radio(chemo)therapy has the potential to predict LRR. In particular, the changes in the tumour hypoxia during the first two weeks of the treatment may be used for adaptive treatment approaches.

## Declaration of Competing Interest

The authors declare that they have no known competing financial interests or personal relationships that could have appeared to influence the work reported in this paper.
