# Supplementary material for: Evolution of the hypoxic compartment on sequential oxygen partial pressure maps during radiochemotherapy in advanced head and neck cancer
Source: Phys Imaging Radiat Oncol. 2021 Feb 11;17:100–5. doi: 10.1016/j.phro.2021.01.011 (PMC8058025; doi:10.1016/j.phro.2021.01.011)
Supplement: Supplementary Data 1 [file mmc1.docx]

Table A: Loco-regional recurrence status (LRR, where 1 indicates that the patient developed loco-regional recurrence) and follow-up time (in months) for the patient data set.

| **N** | **LRR** | **Follow-up time [months]** |
| --- | --- | --- |
| 1 | 0 | 49 |
| 2 | 1 | 10 |
| 3 | 1 | 7 |
| 4 | 0 | 41 |
| 5 | 1 | 7 |
| 6 | 1 | 8 |
| 7 | 1 | 3 |
| 8 | 1 | 14 |
| 9 | 0 | 23 |
| 10 | 0 | 24 |
| 11 | 0 | 19 |
| 12 | 1 | 13 |
| 13 | 0 | 60 |
| 14 | 0 | 61 |
| 15 | 1 | 8 |
| 16 | 1 | 11 |
| 17 | 0 | 34 |
| 18 | 1 | 7 |
| 19 | 0 | 44 |
| 20 | 1 | 10 |
| 21 | 0 | 16 |
| 22 | 1 | 7 |
| 23 | 1 | 5 |
| 24 | 0 | 27 |
| 25 | 1 | 10 |
| 26 | 1 | 8 |
| 27 | 0 | 18 |
| 28 | 0 | 14 |

Table B: Correlation between pO_2_-derived quantities and SUV-derived quantities

| **pO_2_-derived quantity** | **SUV-derived quantity** | | **Spearman’s coefficient** | **p-value** |
| --- | --- | --- | --- | --- |
| SUV_max,1_ | Min pO_2_ | in HTV_1_ | -0.65 | 0.0002 |
| SUV_max,2_ | Min pO_2_ | in HTV_2_ | -0.72 | *<*0.0001 |
| SUV_max,3_ | Min pO_2_ | in HTV_3_ | -0.60 | 0.0008 |
| SUV_max,1_/SUV_background,1_ | Min pO_2_ | in HTV_1_ | -0.99 | *<*0.0001 |
| SUV_max,2_/SUV_background,2_ | Min pO_2_ | in HTV_2_ | -0.96 | *<*0.0001 |
| SUV_max,3_/SUV_background,3_ | Min pO_2_ | in HTV_3_ | -0.99 | *<*0.0001 |
| FMISO-HTV_1_ | pO_2_-HTV_1_ | | 0.79 | *<*0.0001 |
| FMISO-HTV_2_ | pO_2_-HTV_2_ | | 0.69 | 0.0001 |
| FMISO-HTV_3_ | pO_2_-HTV_3_ | | 0.66 | 0.0002 |

Table C: Area Under the Curve (AUC) and corresponding p-value resulting from Receiver Operating Curve (ROC) analysis for pO_2_-derived quantities and corresponding SUV-derived quantities

| **pO_2_-derived quantity** | | **AUC** | **p-value** | **SUV-derived quantity** | **AUC** | **p-value** |
| --- | --- | --- | --- | --- | --- | --- |
| pO_2_-HTV_1_ | | 0.67 | 0.07 | FMISO-HTV_1_ | 0.63 | 0.2 |
| pO_2_-HTV_2_ | | 0.57 | 0.6 | FMISO-HTV_2_ | 0.55 | 0.7 |
| pO_2_-HTV_3_ | | 0.57 | 0.4 | FMISO-HTV_3_ | 0.60 | 0.4 |
| Min pO_2_ | in HTV_1_ | 0.69 | 0.07 | SUV_max,1_ | 0.59 | 0.4 |
| Min pO_2_ | in HTV_2_ | 0.59 | 0.4 | SUV_max,2_ | 0.54 | 0.8 |
| Min pO_2_ | in HTV_3_ | 0.62 | 0.3 | SUV_max,3_ | 0.70 | 0.08 |
| Min pO_2_ | in HTV_1_ | 0.69 | 0.07 | SUV_max,1_/SUV_background,1_ | 0.69 | 0.08 |
| Min pO_2_ | in HTV_2_ | 0.59 | 0.4 | SUV_max,2_/SUV_background,2_ | 0.58 | 0.5 |
| Min pO_2_ | in HTV_3_ | 0.62 | 0.3 | SUV_max,3_/SUV_background,3_ | 0.62 | 0.3 |
| Slope of pO_2_-HTV(t) | | 0.75 | **0.007** | Slope of FMISO-HTV(t) | 0.60 | 0.4 |
| pO_2_ (HTV_1_-HTV_2_) | | 0.73 | **0.02** | FMISO (HTV_1_-HTV_2_) | 0.67 | 0.1 |
| pO_2_ (HTV_1_-HTV_3_) | | 0.73 | **0.02** | FMISO (HTV_1_-HTV_3_) | 0.58 | 0.5 |
| pO_2_ (HTV_2_-HTV_3_) | | 0.52 | 0.9 | FMISO (HTV_2_-HTV_3_) | 0.53 | 0.8 |
| pO2 %HTV_1_*_,_*_2_ | | 0.75 | **0.04** | FMISO %HTV_1_*_,_*_2_ | 0.57 | 0.6 |
| pO2 %HTV_1_*_,_*_3_ | | 0.53 | 0.8 | FMISO %HTV_1_*_,_*_3_ | 0.55 | 0.7 |
| pO2 %HTV_2_*_,_*_3_ | | 0.65 | 0.2 | FMISO %HTV_2_*_,_*_3_ | 0.52 | 0.9 |
